# Prognostic Value of the Royal Marsden Hospital Score in Patients with De Novo Metastatic Colorectal Cancer Receiving First-Line Systemic Therapy

**DOI:** 10.3390/jcm15187149

**Published:** 2026-09-15

**Authors:** Ebru Çiçek, Zeliha Birsin, Mehmet Cem Fidan, Hamza Abbasov, Murat Günaltılı, Emir Çerme, Vali Aliyev, Selin Cebeci, Seda Jeral Evinç, Süheyla Atak, Nebi Serkan Demirci, Özkan Alan

**Affiliations:** Division of Medical Oncology, Department of Internal Medicine, Cerrahpaşa Faculty of Medicine, Istanbul University-Cerrahpaşa, Istanbul 34320, Türkiyedktr.aliyev@gmail.com (V.A.);

**Keywords:** metastatic colorectal cancer, Royal Marsden Hospital score, prognosis, survival

## Abstract

**Background:** The Royal Marsden Hospital (RMH) score is a simple prognostic score based on serum albumin, lactate dehydrogenase, and the number of metastatic organ sites. Although its prognostic value has been demonstrated across several advanced cancer populations, evidence specifically in patients with de novo metastatic colorectal cancer (mCRC) initiating first-line treatment remains limited. **Methods**: This single-center retrospective cohort study included 189 patients with de novo mCRC who initiated first-line systemic treatment between January 2015 and January 2026. Patients were classified as low risk (RMH score 0–1) or high risk (RMH score 2–3). Progression-free survival (PFS) and overall survival (OS) were estimated using the Kaplan–Meier method, and factors independently associated with survival outcomes were evaluated using multivariable Cox regression. **Results**: Of the 189 patients, 142 (75.1%) were classified as low risk and 47 (24.9%) as high risk. Median PFS and OS for the entire cohort were 11.3 and 27.4 months, respectively. Median PFS was 11.8 months in the low-risk group and 7.6 months in the high-risk group (*p* < 0.001). Median OS was 33.8 and 13.7 months, respectively (*p* < 0.001). After adjustment for relevant clinicopathological factors, high-risk RMH status remained independently associated with shorter PFS (HR, 2.16; 95% CI, 1.51–3.09; *p* < 0.001) and OS (HR, 2.95; 95% CI, 2.03–4.29; *p* < 0.001). **Conclusions:** The RMH score was independently associated with both PFS and OS in patients with de novo mCRC initiating first-line systemic treatment. Based on simple and routinely available baseline parameters, the RMH score may serve as a complementary prognostic marker alongside established clinical and molecular factors.

## 1. Introduction

Colorectal cancer continues to pose a major global health challenge, ranking third in incidence and second in cancer-related mortality worldwide [1]. Approximately 15–30% of patients are diagnosed with de novo metastatic colorectal cancer (mCRC), with the liver, lungs, lymph nodes, and peritoneum being the most common sites of metastasis [2]. According to current guidelines, mCRC is considered a heterogeneous disease that requires different treatment approaches depending on molecular features, primary tumor location, disease extent, and patient-related clinical characteristics. Current treatment options include immunotherapy, cytotoxic chemotherapy, and targeted biological agents, including anti-vascular endothelial growth factor (anti-VEGF) agents and anti-epidermal growth factor receptor (anti-EGFR) antibodies [3]. Despite advances in treatment, significant differences in treatment response and survival persist among patients, with median overall survival (OS) in mCRC currently reported to be approximately 25–35 months [4]. Therefore, identifying prognostic factors that affect disease course and survival is clinically important.

In mCRC, prognosis is influenced not only by patient- and tumor-related characteristics but also by disease extent, molecular markers, and routine laboratory findings [5]. In this context, various laboratory parameters, including serum bilirubin, albumin, alkaline phosphatase, lactate dehydrogenase (LDH), white blood cell count, hemoglobin, platelet count, and absolute neutrophil count, have been investigated for their association with survival outcomes [5,6]. LDH is a key enzyme involved in glycolytic metabolism, and elevated serum levels may reflect tumor hypoxia, increased metabolic activity, greater tumor burden, and more aggressive tumor biology. Consistent with these findings, a meta-analysis demonstrated that elevated serum LDH levels were associated with shorter OS in patients with mCRC [7,8]. Serum albumin reflects not only nutritional status but also the systemic inflammatory response in patients with cancer. During tumor-related inflammation, increased cytokine activity may reduce albumin synthesis, while inadequate nutritional intake associated with advanced disease may further contribute to the development of hypoalbuminemia. Low serum albumin levels have been associated with adverse clinical outcomes and shorter survival across various malignancies [9,10].

Beyond individual laboratory parameters, prognostic models and inflammation-based markers have also been investigated in mCRC. The GERCOR model, developed in previously untreated patients with mCRC, incorporates performance status and LDH to stratify patients into prognostic risk groups [11]. The neutrophil-to-lymphocyte ratio (NLR), a readily available marker of systemic inflammation, has also been extensively evaluated for its association with survival in CRC [12]. These findings suggest that prognosis in mCRC is influenced by multiple clinical and laboratory factors and that composite prognostic scores integrating these variables may provide a more comprehensive approach to risk stratification.

Among these composite prognostic scores, the Royal Marsden Hospital (RMH) score is a simple and easily applicable prognostic scoring system comprising three objective and routinely available clinical and laboratory parameters: serum albumin level, serum LDH level, and the number of metastatic organ sites. It was originally developed to estimate prognosis and support the selection of patients with advanced cancer being considered for phase I clinical trials. Subsequent studies have demonstrated the prognostic value of the RMH score across different tumor types and clinical settings, with higher scores being associated with shorter OS and progression-free survival (PFS) [13,14]. However, evidence regarding its prognostic utility in patients with de novo mCRC remains limited, particularly among those receiving first-line treatment in routine clinical practice. Therefore, we investigated the association between the RMH score and survival outcomes in this patient population.

## 2. Materials and Methods

### 2.1. Study Design and Population

**Figure 1:**
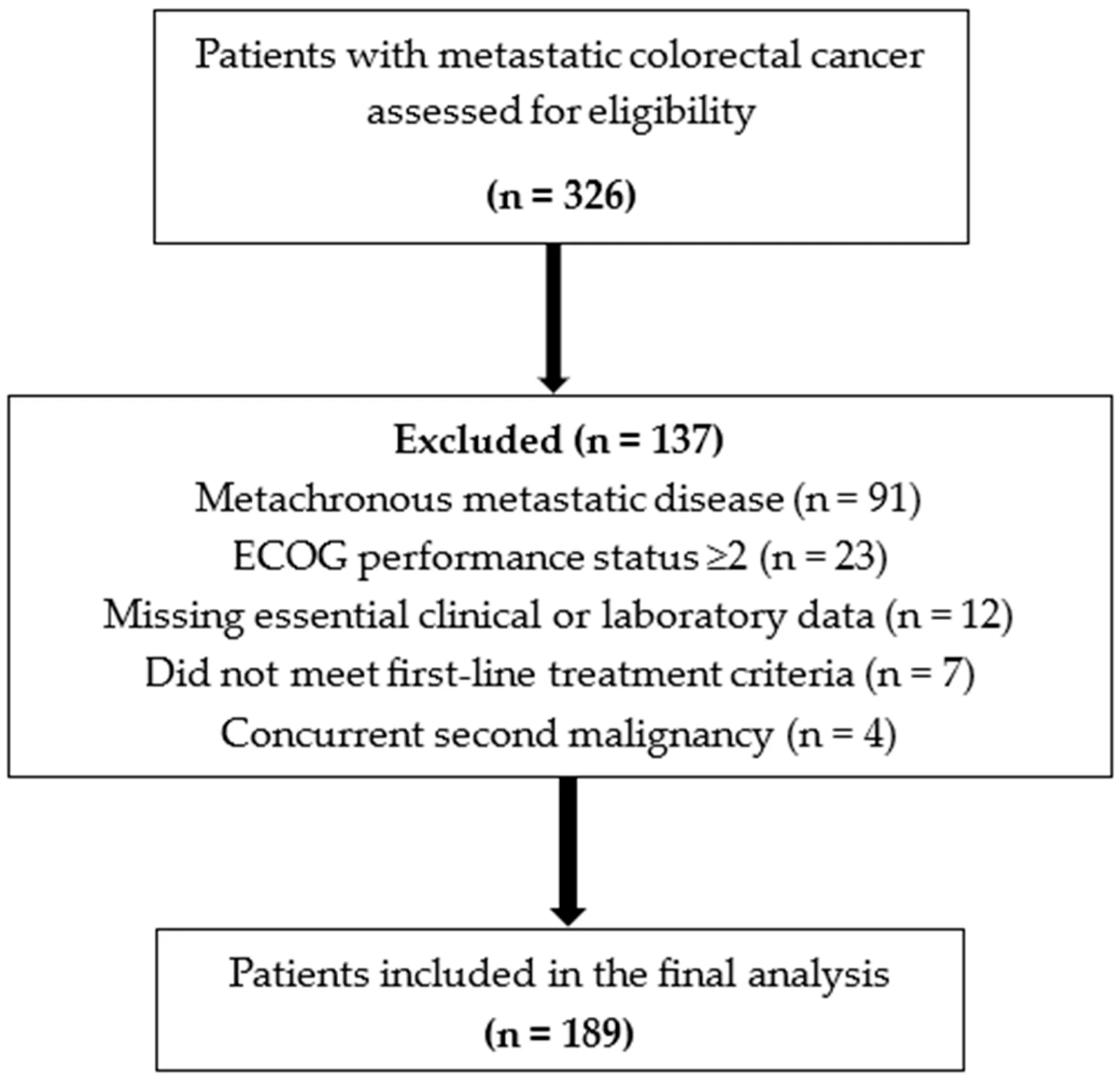
Flow diagram of patient selection.

This single-center retrospective cohort study included patients with histopathologically confirmed de novo mCRC who were treated at our center between January 2015 and January 2026. The inclusion criteria were as follows: (1) age ≥ 18 years; (2) histopathologically confirmed colorectal cancer with metastatic disease at initial diagnosis; (3) Eastern Cooperative Oncology Group (ECOG) performance status of 0–1 at the initiation of first-line systemic therapy; (4) receipt of first-line fluoropyrimidine-based doublet chemotherapy combined with either oxaliplatin or irinotecan, with or without an anti-VEGF or anti-EGFR agent; and (5) availability of serum albumin and LDH levels measured within 14 days before the initiation of first-line systemic therapy. None of the patients, including the nine patients with deficient mismatch repair (dMMR) tumors, received first-line immune checkpoint blockade. The exclusion criteria were as follows: (1) age younger than 18 years; (2) colorectal cancer not classified as stage IV at initial diagnosis; (3) ECOG performance status ≥ 2; (4) missing essential clinical or laboratory data; and (5) presence of a concurrent second malignancy. The patient selection process is summarized in Figure 1.

### 2.2. Data Collection, Study Variables, and Outcome Definitions

Patient data were retrospectively collected from archived patient files and electronic medical records. Data collected for each patient included demographic characteristics (age and sex), comorbidities, ECOG performance status, primary tumor location, RAS mutation status, histological subtype and grading information, first-line treatment regimen, and the number and location of metastatic organ sites. Histological data were extracted directly from the original pathology reports without reassessment. RAS and BRAF mutation status were determined using polymerase chain reaction-based molecular assays. Mismatch repair (MMR) status was assessed by immunohistochemistry, while HER2 status was initially evaluated by immunohistochemistry and, when indicated, confirmed by silver in situ hybridization. Baseline laboratory parameters, including serum LDH, albumin, absolute neutrophil count, and absolute lymphocyte count, were obtained within 14 days before the initiation of first-line systemic therapy. Values obtained during periods of active infection were not used, and the closest available value measured in the absence of active infection within this period was selected. The number and location of metastatic organ sites were determined from baseline staging imaging, primarily contrast-enhanced CT of the chest, abdomen, and pelvis; additional imaging modalities, including MRI or PET/CT, were reviewed when clinically indicated and available.

The RMH score was calculated at baseline using serum albumin and LDH levels and the number of metastatic organ sites, as defined in Table 1. One point was assigned for each of the following: serum albumin < 3.5 g/dL, LDH above the institutional upper limit of normal (>250 U/L), and involvement of ≥3 metastatic organ sites, resulting in a total score ranging from 0 to 3. Patients were subsequently classified into two risk groups: low risk (RMH score 0–1) and high risk (RMH score 2–3).

The simplified GERCOR prognostic classification was calculated using ECOG performance status and serum LDH level [11]. According to the original classification, ECOG performance status of 0, 1, and 2 is assigned to 0, 1, and 2 points, respectively, and 2 additional points are assigned for an LDH level above the upper limit of normal. Patients are classified as low risk (score 0), intermediate risk (scores 1–2), or high risk (scores 3–4). Because the present cohort included only patients with ECOG performance status 0–1, observed GERCOR scores ranged from 0 to 3. The neutrophil-to-lymphocyte ratio (NLR) was calculated by dividing the absolute neutrophil count by the absolute lymphocyte count.

The primary endpoints of the study were PFS and OS. PFS was defined as the time from the initiation of first-line systemic therapy to radiologically documented disease progression or death from any cause, whichever occurred first. Patients were followed according to routine institutional clinical practice, with radiological assessments generally performed at 8–12-week intervals. Disease progression was determined based on radiological evidence of tumor progression on routine follow-up imaging. OS was defined as the time from the start of treatment to death from any cause. Patients without the relevant event were censored at the date of their last follow-up. The data cutoff date was 11 June 2026.

### 2.3. Ethical Considerations

This study was conducted in accordance with the principles of the Declaration of Helsinki. The study protocol was approved by the Ethics Committee of Istanbul University-Cerrahpaşa, Cerrahpaşa Faculty of Medicine (approval number: 2026/297). Due to the retrospective nature of the study, the requirement for informed consent was waived by the ethics committee, and all patient data were anonymized before analysis.

### 2.4. Statistical Analysis

Data were analyzed using IBM SPSS Statistics, version 26.0 (IBM Corp., Armonk, NY, USA). The normality of continuous variables was tested using the Kolmogorov–Smirnov test, Q–Q plots, and histograms. Variables with a normal distribution were compared between groups using the independent-samples *t*-test, whereas variables without a normal distribution were compared using the Mann–Whitney U test. Comparisons of categorical variables between groups were performed using the chi-square test or Fisher’s exact test, as appropriate. Continuous variables were presented as median (minimum–maximum), whereas categorical variables were presented as number and percentage [n (%)].

PFS and OS were estimated using the Kaplan–Meier method, and survival differences between groups were assessed using the log-rank test. To identify prognostic factors associated with PFS and OS, univariable Cox regression analyses were initially performed.

Variables with a *p*-value < 0.20 in univariable analyses or those considered clinically relevant were subsequently included in the multivariable Cox regression model using the backward likelihood ratio method to obtain a more parsimonious final model. In addition to the reduced models, full multivariable Cox models were fitted by simultaneously entering clinically relevant covariates, including age, ECOG performance status, primary tumor location, RAS status, peritoneal involvement, liver metastasis, first-line chemotherapy regimen, targeted therapy, and RMH risk group, irrespective of their univariable significance. Interactions between RMH risk group and targeted therapy, RAS status, and primary tumor location were additionally evaluated by adding each interaction term separately to the full multivariable Cox models. To account for potential temporal changes over the study period, a sensitivity analysis was performed by additionally adjusting the full multivariable Cox models for the period of metastatic diagnosis (before 2020 vs. 2020 onwards). NLR was analyzed as a continuous variable, with hazard ratios expressed per 1-standard-deviation (SD) increase. The proportional hazards assumption was assessed using Schoenfeld residuals. The GERCOR classification was evaluated separately as a comparator prognostic model and was not included in the RMH-containing multivariable model because of overlapping components between the two prognostic systems.

The discriminative performance of the dichotomized RMH risk group (low vs. high), GERCOR classification, and NLR for PFS and OS was assessed using Harrell’s concordance index (C-index) calculated with the concordance() function in the survival package, with 95% confidence intervals derived using bootstrap resampling. Pairwise differences in C-index between the RMH risk group and each comparator were evaluated using paired concordance estimates in R version 4.6.1 (R Foundation for Statistical Computing, Vienna, Austria). To assess the incremental prognostic value of the RMH risk group, a baseline clinical model was compared with an extended model additionally including the dichotomized RMH risk group. Changes in Harrell’s C-index (ΔC-index) were estimated, with 95% confidence intervals derived from 1000 bootstrap resamples, and the nested models were compared using the likelihood ratio test. Time-dependent areas under the curve (AUCs) were additionally estimated at 12 and 24 months for PFS and OS. A two-sided *p*-value < 0.05 was considered statistically significant.

## 3. Results

### 3.1. Baseline Characteristics

A total of 189 patients with de novo mCRC were included in this study. The median age was 62 years (range, 21–85), and 41.8% of patients were aged ≥65 years. Male patients accounted for 60.8% of the cohort, and 40.7% had at least one comorbid condition. The left colon was the most common primary tumor site (46.0%), and conventional adenocarcinoma was the predominant histological subtype (87.3%). The liver was the most common metastatic site (72.0%). BRAF, MMR, and HER2 status was assessed in 147, 150, and 38 patients, respectively; these molecular data were unavailable in the remaining patients. BRAF mutations were identified in 2 patients, dMMR in 9 patients, and HER2 positivity in 1 patient. Based on the RMH score, 142 patients (75.1%) were classified as low risk and 47 patients (24.9%) were classified as high risk.

Baseline clinicopathological and treatment characteristics according to RMH risk group are summarized in Table 2. Patients in the high-risk RMH group were more frequently classified as ECOG PS 1 than those in the low-risk group (78.7% vs. 50.0%, *p* = 0.001) and had a higher median NLR (4.40 vs. 2.83, *p* < 0.001). No statistically significant between-group differences were observed in age, sex, comorbidity status, primary tumor location, RAS mutation status, histological subtype, grading category, first-line chemotherapy regimen, targeted therapy, or the presence of liver or peritoneal metastases. The distribution of individual RMH score components and score categories is presented in Table 3.

### 3.2. Progression-Free Survival

**Figure 2:**
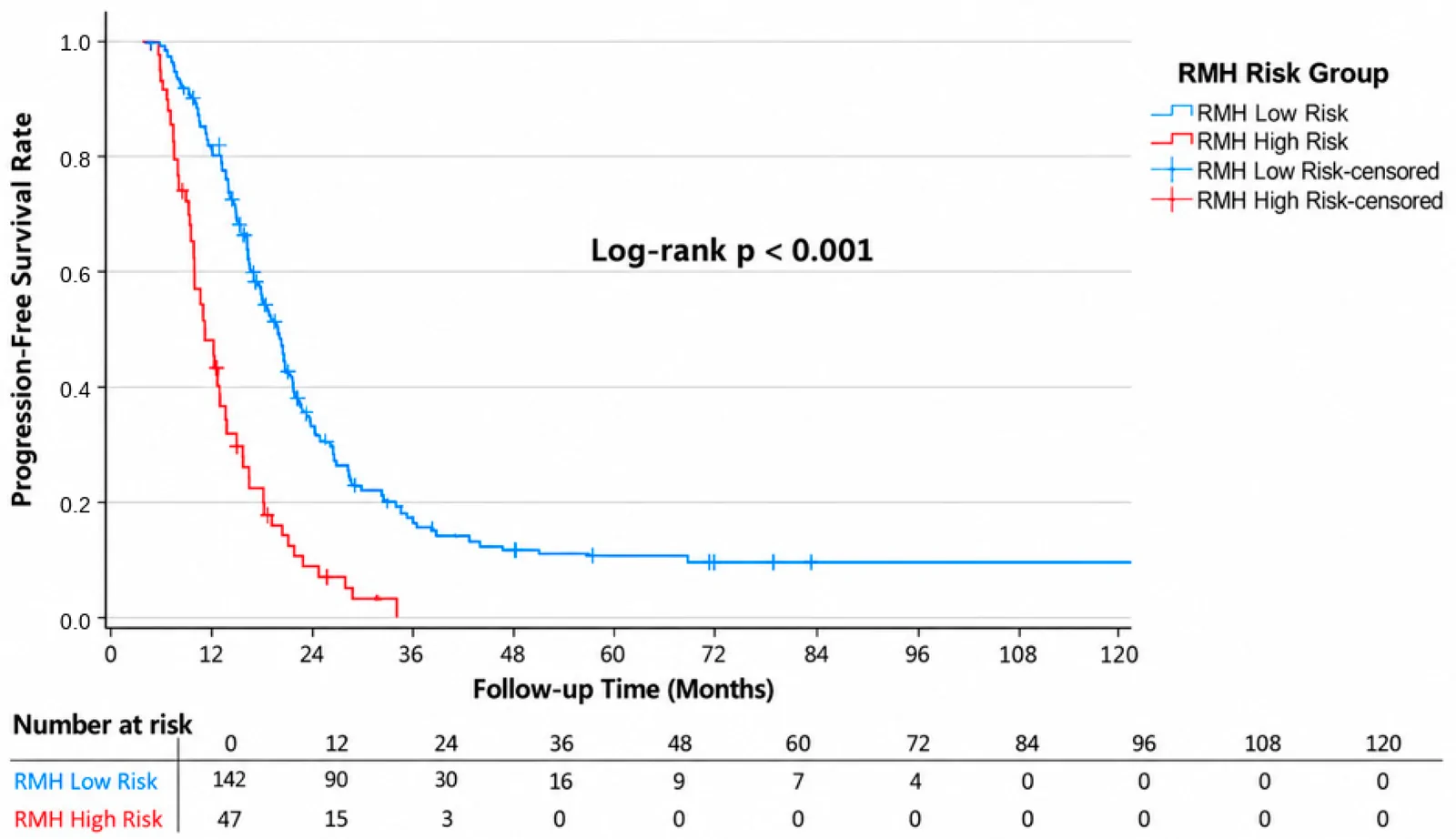
Progression-Free Survival. Kaplan–Meier survival curves comparing patients in the RMH low-risk and high-risk groups. Censored observations are indicated on the curves.

**Figure 3:**
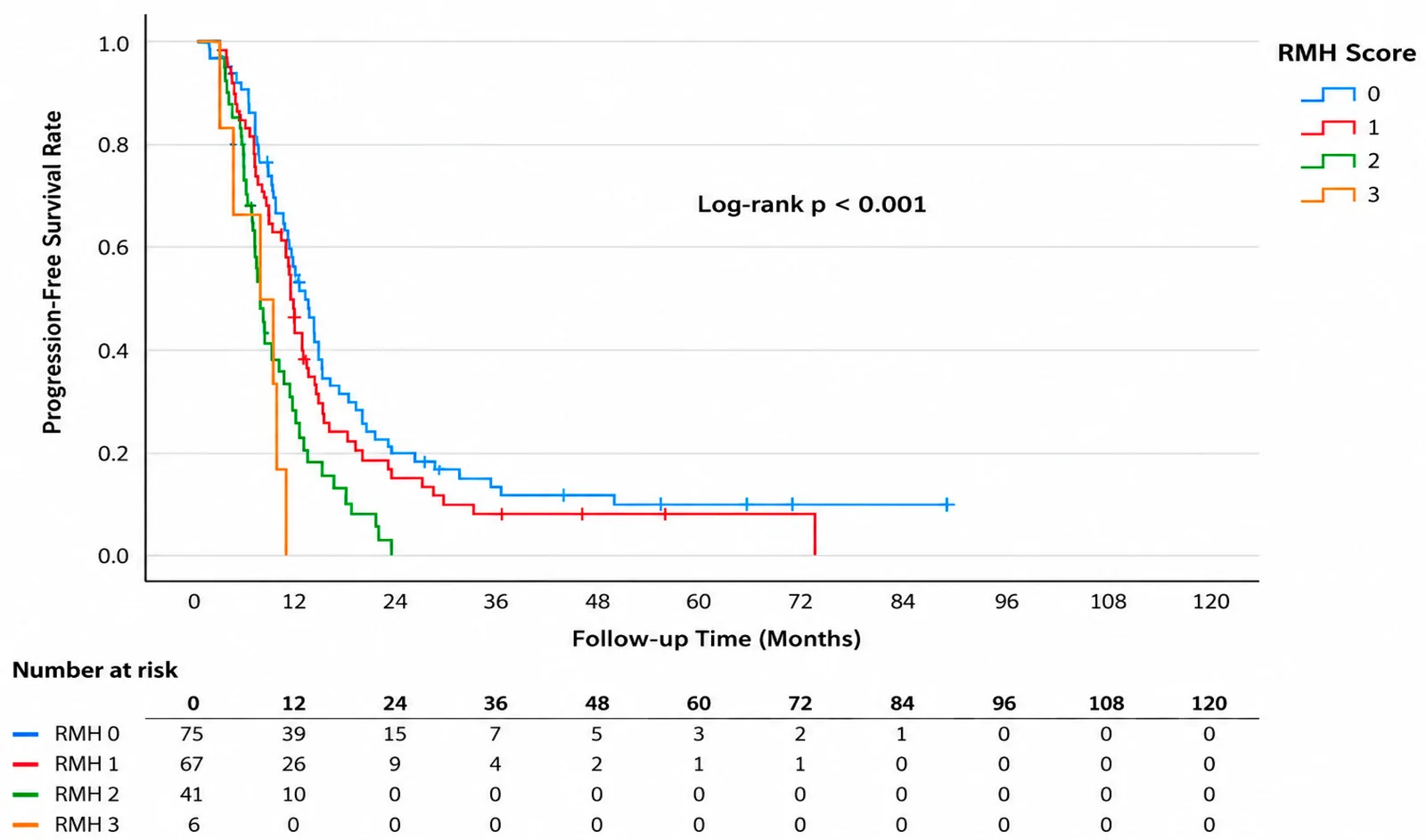
Progression-Free Survival According to Individual RMH Scores. Kaplan–Meier survival curves according to RMH scores of 0, 1, 2, and 3. Censored observations are indicated on the curves.

At the data cutoff, the median follow-up duration, calculated using the reverse Kaplan–Meier method, was 67.2 months (95% CI, 53.0–81.5). During follow-up, 166 patients (87.8%) experienced disease progression, and 142 (75.1%) died. Median PFS for the overall cohort was 11.3 months (95% CI, 10.4–12.2). Median PFS was 11.8 months (95% CI, 10.6–13.1) in the RMH low-risk group versus 7.6 months (95% CI, 6.7–8.5) in the RMH high-risk group, with a statistically significant difference in PFS between the two groups (*p* < 0.001, log-rank test) (Figure 2). PFS was further evaluated across the four individual RMH score categories. Median PFS was 13.3 months (95% CI, 11.2–15.4), 11.4 months (95% CI, 10.5–12.3), 7.7 months (95% CI, 6.6–8.7), and 7.6 months (95% CI, 1.6–13.7) for RMH scores of 0, 1, 2, and 3, respectively, demonstrating a significant difference across the four groups (overall log-rank *p* < 0.001) (Figure 3). When the RMH score was analyzed as an ordinal variable, each 1-point increase was associated with a higher risk of progression or death (HR, 1.52; 95% CI, 1.26–1.83; *p* < 0.001). Individual component analyses are provided in Supplementary Table S3.

In univariable Cox regression analysis, high RMH risk was associated with shorter PFS (HR, 2.13; 95% CI, 1.50–3.02; *p* < 0.001). NLR was not significantly associated with PFS (HR per 1-SD increase, 1.12; 95% CI, 0.95–1.33; *p* = 0.145). For the GERCOR classification, high-risk patients had significantly shorter PFS than low-risk patients (HR, 1.57; 95% CI, 1.04–2.38; *p* = 0.032), whereas the intermediate-risk group did not differ significantly from the low-risk group (HR, 1.23; 95% CI, 0.84–1.79; *p* = 0.281; overall *p* = 0.097). Variables with *p*-values < 0.20 in the univariable analysis, including age (*p* = 0.117), histology (*p* = 0.077), peritoneal metastasis (*p* = 0.128), and first-line targeted therapy (*p* = 0.187), as well as NLR (*p* = 0.145), were included in the multivariable Cox regression model. RAS mutation status (*p* = 0.208) was also included because of its established prognostic and predictive relevance in metastatic colorectal cancer. GERCOR was analyzed separately and was not included in the RMH-containing multivariable model because both scores incorporate LDH. In multivariable Cox regression analysis, high RMH risk remained independently associated with shorter PFS (HR, 2.16; 95% CI, 1.51–3.09; *p* < 0.001) (Table 4). In the full multivariable Cox model described in the Methods, high RMH risk was also associated with shorter PFS (HR, 2.24; 95% CI, 1.53–3.26; *p* < 0.001; Supplementary Table S1). Additional adjustment for the period of metastatic diagnosis (before 2020 versus 2020 onwards) yielded a similar estimate (HR, 2.28; 95% CI, 1.55–3.34; *p* < 0.001). No statistically significant interactions were observed between RMH risk group and targeted therapy (*p* = 0.617), RAS status (*p* = 0.473), or primary tumor location (*p* = 0.573) for PFS. Schoenfeld residual tests showed no evidence of violation of the proportional-hazards assumption (global *p* = 0.196; RMH *p* = 0.996).

For PFS, the C-index was 0.574 (95% CI, 0.539–0.608) for the dichotomized RMH risk groups, 0.550 (95% CI, 0.504–0.597) for GERCOR, and 0.551 (95% CI, 0.500–0.602) for NLR. The C-index was numerically higher for RMH, but the differences versus GERCOR and NLR were not statistically significant (*p* = 0.244 and *p* = 0.432, respectively). In the incremental-value analysis described in the Methods, the baseline clinical model was compared with the extended model including the RMH risk group. The addition of RMH increased the C-index from 0.564 to 0.610 (ΔC-index = 0.046), with a bootstrap mean ΔC-index of 0.040 (95% CI, 0.005–0.084). Model fit also improved significantly (likelihood-ratio χ^2^ = 15.84, df = 1, *p* < 0.001). The 12-month time-dependent AUC increased from 0.572 to 0.631 (*p* = 0.102), while the 24-month AUC increased from 0.629 to 0.738 (*p* = 0.005).

### 3.3. Overall Survival

**Figure 4:**
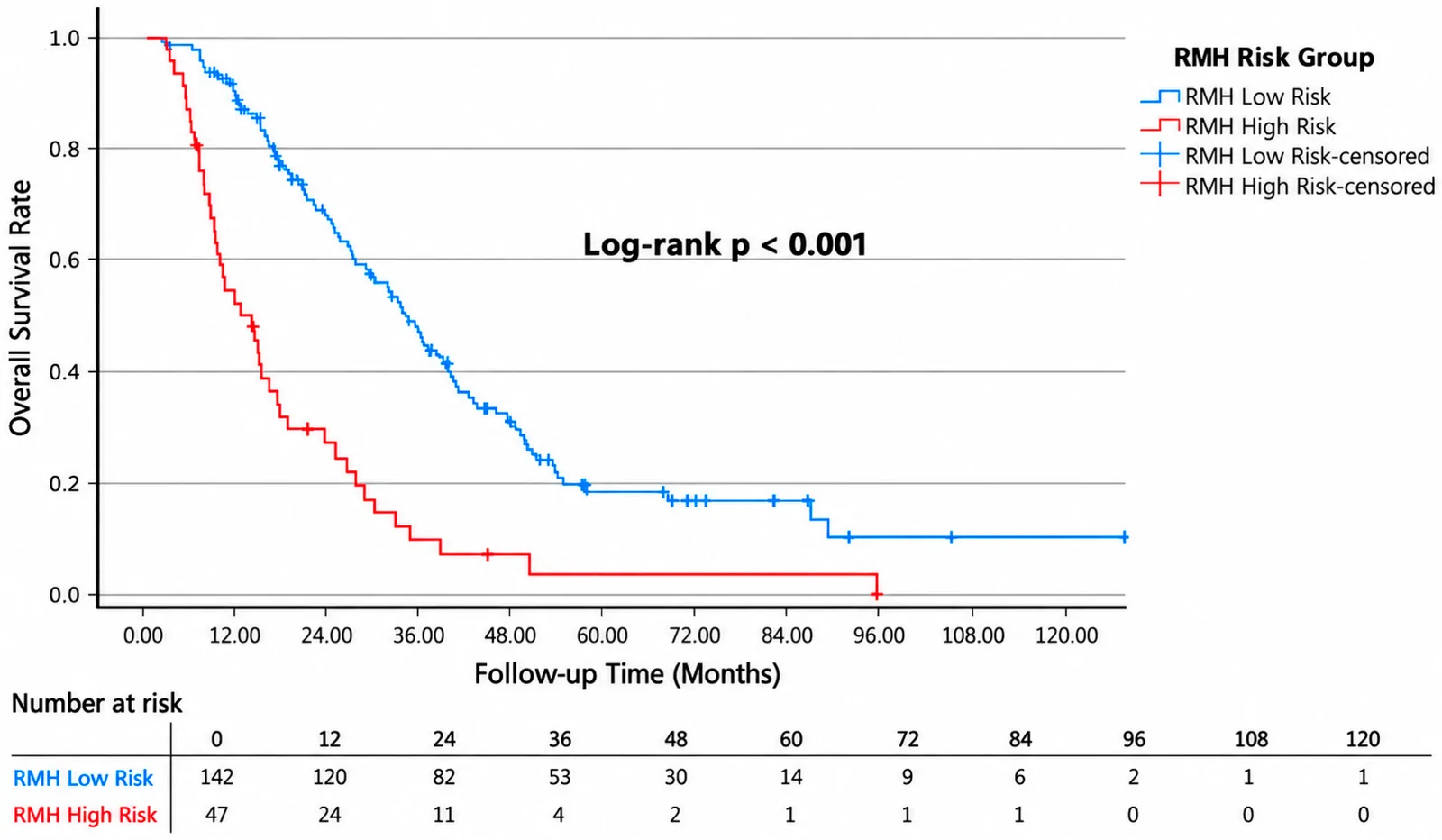
Overall Survival. Kaplan–Meier survival curves comparing patients in the RMH low-risk and high-risk groups. Censored observations are indicated on the curves, and numbers at risk are shown below the plot.

**Figure 5:**
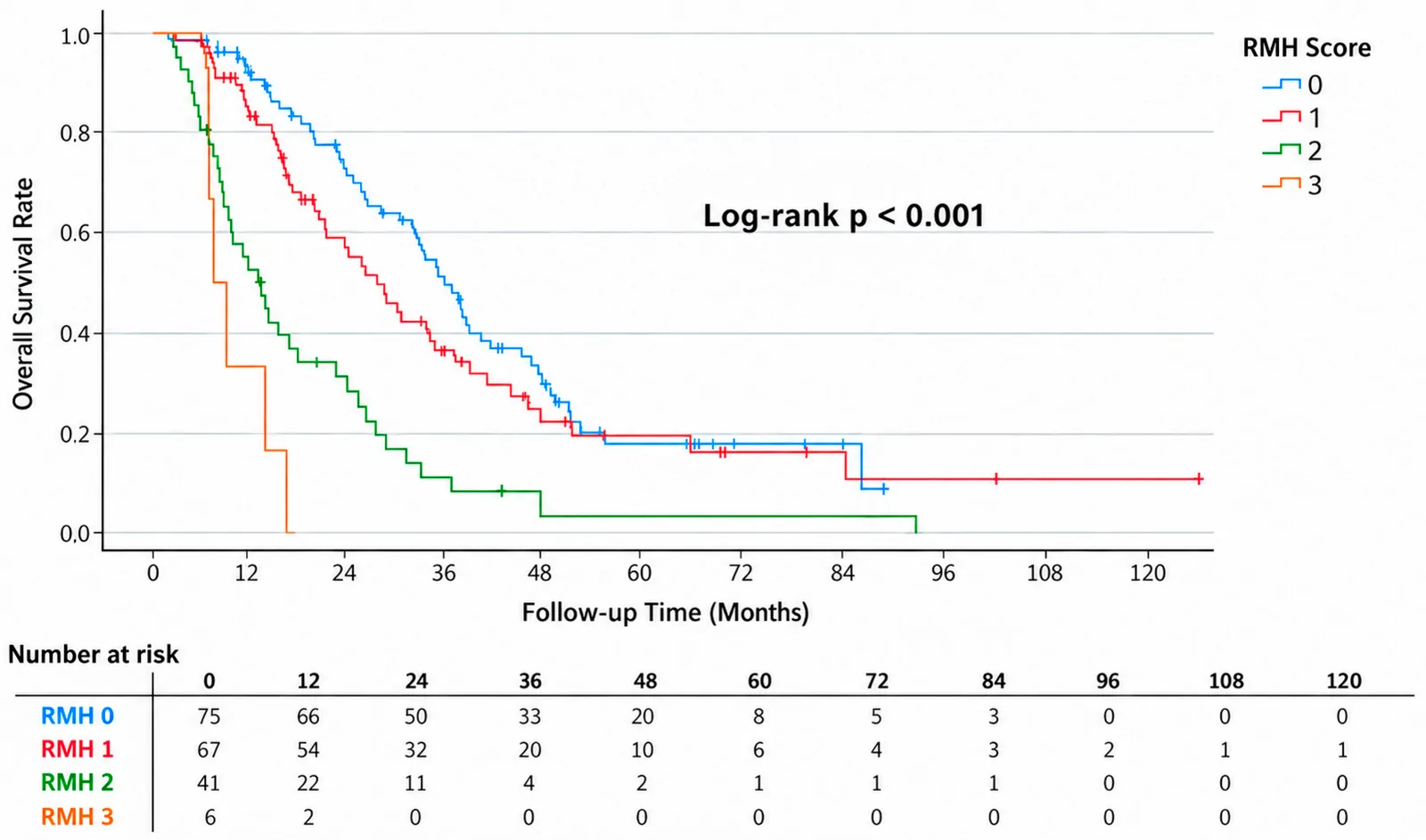
Overall Survival According to Individual RMH Scores. Kaplan–Meier survival curves according to RMH scores of 0, 1, 2, and 3. Censored observations are indicated on the curves, and numbers at risk are shown below the plot.

Median OS in the overall cohort was 27.4 months (95% CI, 22.7–32.0). Median OS was 33.8 months (95% CI, 29.8–37.8) in the RMH low-risk group versus 13.7 months (95% CI, 8.7–18.7) in the RMH high-risk group, with a statistically significant difference in OS between the two groups (*p* < 0.001, log-rank test) (Figure 4). OS was further evaluated across the four individual RMH score categories. Median OS was 37.7 months (95% CI, 32.3–43.1), 28.7 months (95% CI, 21.4–36.1), 14.2 months (95% CI, 10.0–18.4), and 7.6 months (95% CI, 4.9–10.3) for RMH scores of 0, 1, 2, and 3, respectively, demonstrating a significant difference across the four groups (overall log-rank *p* < 0.001) (Figure 5). When the RMH score was analyzed as an ordinal variable, each 1-point increase was associated with a higher risk of death (HR, 1.81; 95% CI, 1.47–2.23; *p* < 0.001). Individual component analyses are provided in Supplementary Table S3.

In univariable Cox regression analysis, age ≥ 65 years (*p* = 0.005), ECOG PS 1 (*p* = 0.032), RAS-mutant status (*p* = 0.023), the presence of peritoneal metastasis *(p* = 0.044), and high RMH risk (HR, 3.01; 95% CI, 2.08–4.36; *p* < 0.001) were significantly associated with shorter OS. NLR was not significantly associated with OS (HR per 1-SD increase, 1.15; 95% CI, 0.98–1.36; *p* = 0.087). For the GERCOR classification, high-risk patients had significantly shorter OS than low-risk patients (HR, 1.86; 95% CI, 1.19–2.90; *p* = 0.006), whereas the intermediate-risk group did not differ significantly from the low-risk group (HR, 1.28; 95% CI, 0.85–1.92; *p* = 0.240; overall *p* = 0.020). Significant factors identified in the univariable analysis, together with histology (*p* = 0.128) and NLR (*p* = 0.087), were included in the multivariable Cox regression model based on the prespecified threshold of *p* < 0.20. GERCOR was analyzed separately and was not included in the RMH-containing multivariable model because both scores incorporate LDH. In multivariable analysis, high RMH risk remained independently associated with shorter OS (HR, 2.95; 95% CI, 2.03–4.29; *p* < 0.001), while age ≥ 65 years also remained statistically significant (HR, 1.59; 95% CI, 1.13–2.25; *p* = 0.008) (Table 5). In the full multivariable Cox model described in the Methods, high RMH risk was also associated with shorter OS (HR, 3.40; 95% CI, 2.23–5.18; *p* < 0.001; Supplementary Table S2). Additional adjustment for the period of metastatic diagnosis (before 2020 versus 2020 onwards) yielded a similar estimate (HR, 3.39; 95% CI, 2.22–5.16; *p* < 0.001). No statistically significant interactions were observed between RMH risk group and targeted therapy (*p* = 0.059), RAS status (*p* = 0.710), or primary tumor location (*p* = 0.869) for OS. The global Schoenfeld residual test did not indicate a significant violation of the proportional-hazards assumption (*p* = 0.182); however, the variable-specific test suggested possible non-proportionality for the RMH risk group (*p* = 0.033). An additional analysis incorporating an RMH × log(time) interaction showed that the interaction term did not reach statistical significance (*p* = 0.052), although a time-varying effect could not be excluded.

For OS, the C-index was 0.621 (95% CI, 0.582–0.661) for the dichotomized RMH risk groups, 0.592 (95% CI, 0.543–0.641) for GERCOR, and 0.561 (95% CI, 0.504–0.618) for NLR. The C-index was numerically higher for RMH, but the differences versus GERCOR and NLR were not statistically significant (*p* = 0.153 and *p* = 0.056, respectively). In the incremental-value analysis described in the Methods, the addition of RMH to the baseline clinical model increased the C-index from 0.634 to 0.692 (ΔC-index = 0.058), with a bootstrap mean ΔC-index of 0.054 (95% CI, 0.018–0.095). Model fit also improved significantly according to the likelihood-ratio test (χ^2^ = 29.58, df = 1, *p* < 0.001). The 12-month time-dependent AUC increased from 0.700 to 0.774 (*p* = 0.088), and the 24-month AUC increased from 0.716 to 0.764 (*p* = 0.160); however, neither difference reached statistical significance.

## 4. Discussion

This retrospective cohort study of 189 patients with de novo mCRC demonstrated a significant association between the RMH score and survival outcomes, with high RMH risk being associated with shorter PFS and OS. These findings extend the prognostic relevance of the RMH score to patients with de novo mCRC at the initiation of first-line systemic therapy.

The RMH score was originally developed in 212 patients with advanced cancer enrolled in phase I clinical trials and was later prospectively validated in a cohort of 78 patients. In the validation cohort, median OS was 33.0 weeks in the low-risk group and 15.7 weeks in the high-risk group [13]. The prognostic relevance of the RMH score has since been demonstrated in different malignancies, including CRC, lung cancer, and sarcoma [15,16,17]. A 2024 systematic review and meta-analysis of 19 studies involving 127,230 patients further showed that a high RMH score was associated with shorter OS (HR, 2.09; 95% CI, 1.87–2.33) and PFS (HR, 1.80; 95% CI, 1.48–2.18) [14]. More recent studies have reported similar findings in melanoma and renal cell carcinoma [18,19]. Consistent with these previous studies, high RMH risk status was also associated with poorer survival outcomes in our cohort.

CRC-specific evidence regarding the prognostic value of the RMH score remains limited and has largely been derived from early-phase clinical trial populations. Hong et al. evaluated 144 patients with advanced CRC referred to a phase I clinic and demonstrated that a high RMH score was independently associated with shorter OS. However, patients in this cohort had received a median of four prior lines of systemic therapy and therefore represented a heavily pretreated population at a later point in the metastatic disease course [15]. This may limit the generalizability of these findings to treatment-naïve patients beginning first-line therapy. In contrast, our study evaluated the association between the RMH score and survival at an earlier and clinically distinct point in the metastatic disease course, among patients with de novo mCRC initiating first-line systemic therapy.

The prognostic value of the RMH score may be explained by its integration of complementary patient- and disease-related characteristics. Serum albumin reflects nutritional and inflammatory status, LDH is associated with tumor metabolic activity and biological aggressiveness, and the number of metastatic organ sites reflects disease burden. Previous studies have demonstrated the prognostic significance of hypoalbuminemia, elevated LDH levels, and metastatic burden in colorectal cancer and other malignancies [8,20,21,22,23]. By integrating these complementary parameters into a single score, the RMH score provides a simple assessment of both host condition and tumor biology. This may explain why the RMH score remained independently associated with adverse survival outcomes in our cohort.

The GERCOR model was developed in previously untreated patients with mCRC receiving first-line oxaliplatin- or irinotecan-based chemotherapy. The simplified model, based on performance status and LDH, stratified patients into three prognostic risk groups, with shorter OS in the intermediate- and high-risk groups than in the low-risk group [11]. In our cohort, GERCOR was also significantly associated with OS; however, this separation was mainly observed in the high-risk group (HR, 1.86; 95% CI, 1.19–2.90), whereas no significant difference was observed between the intermediate- and low-risk groups. This may reflect a floor effect, as the exclusion of ECOG PS ≥ 2 patients restricted the observed range of GERCOR scores. Because GERCOR was developed in first-line mCRC patients similar to those included in our study, it provides a relevant model for comparison with the RMH score. The C-index for GERCOR was 0.63 in the original validation cohort and 0.592 in our cohort. RMH showed slightly better discrimination for OS, with a C-index of 0.621, although the difference from GERCOR was not statistically significant. Although the Cox models demonstrated a strong association between the RMH score and survival after multivariable adjustment, the added discrimination provided by the score was limited. Therefore, the RMH score should be considered a complementary prognostic stratifier rather than a standalone tool, and these findings should be regarded as hypothesis-generating.

The prognostic role of pretreatment NLR in CRC has been evaluated in large patient populations. In a meta-analysis of 71 studies by Naszai et al., high NLR was associated with poorer survival outcomes [12]. However, the authors highlighted considerable variation in the NLR cutoff values used across studies and noted that data-driven dichotomization may introduce bias. They therefore suggested that evaluating NLR as a continuous variable may be more appropriate. In a recent retrospective study, continuous dNLR was not independently associated with OS in multivariable analysis [24]. Similarly, in our study, NLR analyzed as a continuous variable was not significantly associated with either PFS or OS, whereas a high RMH score remained independently associated with both outcomes. This difference may be related to the fact that NLR represents a single inflammatory marker, whereas the RMH score combines several prognostic parameters. However, the lack of a statistically significant difference in C-index between RMH and NLR does not support interpreting these findings as evidence of superior prognostic performance of the RMH score.

From a clinical perspective, a key advantage of the RMH score is that it can be readily calculated at the start of treatment using routinely available clinical and laboratory data. Without requiring additional imaging or specialized molecular testing, it may contribute to baseline prognostic assessment. The RMH score should be regarded as a prognostic rather than predictive tool and should not be used to guide the selection of a specific systemic treatment. Instead, it may provide complementary prognostic information alongside established clinical factors, including molecular profile, primary tumor location, performance status, and extent of disease.

One of the main strengths of this study is that it included only patients with de novo mCRC who were evaluated before starting first-line systemic treatment. This provided a more homogeneous clinical setting by assessing patients at a consistent point in the metastatic disease course, before exposure to systemic therapy for metastatic disease. The prognostic value of the RMH score was assessed for both PFS and OS and was further examined in multivariable analyses adjusted for relevant clinicopathological factors. The relatively long follow-up and the use of routine clinical data also support the relevance of the findings to real-world practice.

However, this study has several limitations. The retrospective, single-center design may have introduced selection bias and, together with potential differences in subsequent systemic and local treatments, limited the generalizability of the findings. Although 189 patients were included, the small number of patients with an RMH score of 3 (*n* = 6) limited more detailed subgroup analyses and warrants cautious interpretation of the four-category RMH analysis. In addition, because the study included only patients with de novo metastatic disease and ECOG performance status 0–1 receiving fluoropyrimidine-based first-line chemotherapy, the findings may not be directly generalized to patients with metachronous metastases, poorer performance status, or those receiving alternative first-line approaches such as immunotherapy. Further molecular analyses were limited by the small numbers of patients with BRAF-mutant, dMMR, and HER2-positive tumors, while the absence of data on CEA and nutritional/anthropometric parameters such as BMI, together with missing data for some variables, may have introduced additional selection bias. Finally, the absence of calibration assessment, NRI/IDI analyses, decision-curve analysis, comprehensive internal validation, and external validation limits the ability to fully assess the incremental and clinical utility of the RMH score and to confirm the reproducibility of our findings in independent patient populations.

In conclusion, the RMH score was independently associated with both PFS and OS in patients with de novo mCRC starting first-line systemic treatment. Because it is based on simple and routinely available parameters, the RMH score may provide supplementary prognostic information for baseline risk assessment alongside established clinical and molecular factors. These findings should be confirmed in larger, prospective, multicenter cohorts.

## Figures and Tables

**Table 1:** RMH score calculation and risk classification.

**RMH component**	0 points	1 point
Albumin	≥3.5 g/dL	<3.5 g/dL
LDH ^†^	≤250 U/L	>250 U/L
Metastatic organ sites *	<3	≥3
**Risk classification**	Low risk: 0–1 points	High risk: 2–3 points

^†^ The upper limit of normal for LDH at our institution was 250 U/L. * Multiple metastatic lesions within the same organ were considered as single-organ involvement; only distinct metastatic organ sites were counted.

**Table 2:** Baseline clinicopathological and treatment characteristics according to RMH risk group.

		All Patients	RMH Low-Risk Group	RMH High-Risk Group	*p*-Value
Variables		(*n* = 189)	(*n* = 142)	(*n* = 47)	
Age (years)	Median (min–max)	62 (21–85)	63 (21–85)	62 (33–81)	0.534
Age, n (%)	<65 years	110 (58.2%)	81 (57.0%)	29 (61.7%)	0.575
	≥65 years	79 (41.8%)	61 (43.0%)	18 (38.3%)	
Gender, n (%)	Female	74 (39.2%)	51 (35.9%)	23 (48.9%)	0.113
	Male	115 (60.8%)	91 (64.1%)	24 (51.1%)	
Comorbidity, n (%)	Absent	112 (59.3%)	79 (55.6%)	33 (70.2%)	0.078
	Present	77 (40.7%)	63 (44.4%)	14 (29.8%)	
ECOG PS, n (%)	0	81 (42.9%)	71 (50.0%)	10 (21.3%)	0.001
	1	108 (57.1%)	71 (50.0%)	37 (78.7%)	
Tumor location, n (%)	Right colon	49 (25.9%)	37 (26.1%)	12 (25.5%)	0.953
	Left colon	87 (46.0%)	66 (46.5%)	21 (44.7%)	
	Rectum	53 (28.0%)	39 (27.5%)	14 (29.8%)	
RAS status, n (%)	Wild type	90 (47.6%)	72 (50.7%)	18 (38.3%)	0.087
	Mutant	90 (47.6%)	62 (43.7%)	28 (59.6%)	
	Unknown	9 (4.8%)	8 (5.6%)	1 (2.1%)	
Histology subtypes, n (%)	Conv. adenocarcinoma	165 (87.3%)	123 (86.6%)	42 (89.4%)	0.625
	Special subtypes *	24 (12.7%)	19 (13.4%)	5 (10.6%)	
Grading category, n (%) ^†^	1	142 (75.1%)	111 (78.2%)	31 (66.0%)	0.206
	2	40 (21.2%)	27 (19.0%)	13 (27.7%)	
	3	7 (3.7%)	4 (2.8%)	3 (6.4%)	
First-line chemotherapy, n (%)	Oxaliplatin-based	146 (77.2%)	109 (76.8%)	37 (78.7%)	0.781
	Irinotecan-based	43 (22.8%)	33 (23.2%)	10 (21.3%)	
First-line targeted agent, n (%)	Anti-VEGF	120 (63.5%)	90 (63.4%)	30 (63.8%)	0.850
	Anti-EGFR	56 (29.6%)	43 (30.3%)	13 (27.7%)	
	No targeted agent	13 (6.9%)	9 (6.3%)	4 (8.5%)	
Liver metastasis, n (%)		136 (72.0%)	99 (69.7%)	37 (78.7%)	0.234
Peritoneal metastasis, n (%)		43 (22.8%)	29 (20.4%)	14 (29.8%)	0.184
NLR, median (min–max)		3.08 (0.78–20.00)	2.83 (0.78–9.77)	4.40 (0.94–20.00)	<0.001

* Special subtypes included mucinous and signet-ring cell adenocarcinomas. ^†^ For retrospective data harmonization, tumors reported as G1, well differentiated, or low grade were coded as category 1; those reported as G2 or moderately differentiated as category 2; and those reported as G3, poorly differentiated, or high grade as category 3. Abbreviations: Conv., conventional; EGFR, epidermal growth factor receptor; NLR, neutrophil-to-lymphocyte ratio; RMH, Royal Marsden Hospital; VEGF, vascular endothelial growth factor.

**Table 3:** RMH score components and categories.

	All Patients	RMH Low-Risk Group	RMH High-Risk Group
Variables	(*n* = 189)	(*n* = 142)	(*n* = 47)
Albumin < 3.5 g/dL, n (%)	49 (25.9%)	10 (7.0%)	39 (83.0%)
LDH > 250 U/L, n (%)	81 (42.9%)	39 (27.5%)	42 (89.4%)
Metastatic organ sites ≥ 3, n (%)	37 (19.6%)	18 (12.7%)	19 (40.4%)
RMH score, n (%)			
Score 0	75 (39.7%)	75 (52.8%)	—
Score 1	67 (35.4%)	67 (47.2%)	—
Score 2	41 (21.7%)	—	41 (87.2%)
Score 3	6 (3.2%)	—	6 (12.8%)

Abbreviations: LDH, lactate dehydrogenase; RMH, Royal Marsden Hospital.

**Table 4:** Univariable and multivariable Cox regression analyses of factors associated with progression-free survival.

	Univariable Analyses		Multivariable Analyses	
Variable	HR (95% CI)	*p*-Value	HR (95% CI)	*p*-Value
Age (≥65 vs. <65 years)	1.31 (0.93–1.84)	0.117	1.23 (0.88–1.73)	0.223
Gender (male vs. female)	0.85 (0.62–1.16)	0.313	—	—
Comorbidity (present vs. absent)	0.85 (0.62–1.17)	0.338	—	—
ECOG PS (1 vs. 0)	1.21 (0.89–1.65)	0.230	—	—
Histology (special vs. conventional) ^†^	1.50 (0.95–2.36)	0.077	1.54 (0.97–2.46)	0.069
Tumor location		0.637	—	—
Right-sided colon	Reference		—	—
Left-sided colon	0.83 (0.57–1.21)	0.343	—	—
Rectum	0.89 (0.59–1.34)	0.602	—	—
RAS mutation (mutant vs. wild type)	1.22 (0.89–1.66)	0.208	1.10 (0.79–1.53)	0.578
Liver metastasis (yes vs. no)	1.14 (0.80–1.62)	0.457	—	—
Peritoneal metastasis (yes vs. no)	1.33 (0.92–1.93)	0.128	1.07 (0.71–1.59)	0.755
First-line chemotherapy (oxaliplatin vs. irinotecan)	1.02 (0.71–1.45)	0.915	—	—
First-line targeted agent (anti-VEGF vs. anti-EGFR)	0.79 (0.56–1.12)	0.187	1.26 (0.75–2.11)	0.390
NLR (per 1-SD increase)	1.12 (0.95–1.33)	0.145	1.05 (0.76–1.45)	0.790
GERCOR risk group		0.097	—	—
Low	Reference		—	—
Intermediate	1.23 (0.84–1.79)	0.281	—	—
High	1.57 (1.04–2.38)	**0.032**	—	—
RMH risk group (high vs. low)	2.13 (1.50–3.02)	**<0.001**	2.16 (1.51–3.09)	**<0.001**

^†^ Special histological subtypes included mucinous and signet-ring cell adenocarcinomas; conventional refers to conventional adenocarcinoma. The multivariable Cox regression model included 167 patients and 149 events; 13 patients who did not receive targeted therapy and 9 patients with unknown RAS status were excluded from the analysis Abbreviations: CI, confidence interval; EGFR, epidermal growth factor receptor; GERCOR, Groupe Coopérateur Multidisciplinaire en Oncologie; HR, hazard ratio; NLR, neutrophil-to-lymphocyte ratio; RMH, Royal Marsden Hospital; VEGF, vascular endothelial growth factor. Bold values indicate statistically significant findings; blank cells were excluded by backward LR.

**Table 5:** Univariable and multivariable Cox regression analyses of factors associated with overall survival.

	Univariable Analyses		Multivariable Analyses	
Variable	HR (95% CI)	*p*-Value	HR (95% CI)	*p*-Value
Age (≥65 vs. <65 years)	1.62 (1.16–2.28)	**0.005**	1.59 (1.13–2.25)	**0.008**
Gender (male vs. female)	1.05 (0.75–1.48)	0.747	—	—
Comorbidity (present vs. absent)	0.98 (0.70–1.39)	0.949	—	—
ECOG PS (1 vs. 0)	1.45 (1.03–2.03)	**0.032**	1.17 (0.82–1.67)	0.390
Histology (special vs. conventional) ^†^	1.42 (0.90–2.24)	0.128	1.48 (0.93–2.35)	0.103
Tumor location		0.518	—	—
Right-sided colon	Reference		—	—
Left-sided colon	0.85 (0.57–1.27)	0.447	—	—
Rectum	0.77 (0.49–1.20)	0.256	—	—
RAS mutation (mutant vs. wild)	1.48 (1.05–2.07)	**0.023**	1.22 (0.85–1.74)	0.280
Liver metastasis (yes vs. no)	0.88 (0.62–1.27)	0.515	—	—
Peritoneal metastasis (yes vs. no)	1.49 (1.01–2.19)	**0.044**	1.17 (0.78–1.78)	0.449
First-line chemotherapy (oxaliplatin vs. irinotecan)	0.87 (0.59–1.28)	0.497	—	—
First-line targeted agent (anti-VEGF vs. anti-EGFR)	0.86 (0.59–1.25)	0.433	—	—
NLR (per 1-SD increase)	1.15 (0.98–1.36)	**0.087**	0.99 (0.92–1.07)	0.864
GERCOR risk group		**0.020**	—	—
Low	Reference		—	—
Intermediate	1.28 (0.85–1.92)	**0.240**	—	—
High	1.86 (1.19–2.90)	**0.006**	—	—
RMH risk group (high vs. low)	3.01 (2.08–4.36)	**<0.001**	2.95 (2.03–4.29)	**<0.001**

^†^ Special histological subtypes included mucinous and signet-ring cell adenocarcinomas; conventional refers to conventional adenocarcinoma. The multivariable Cox regression model included 167 patients and 124 deaths; 13 patients who did not receive targeted therapy and 9 patients with unknown RAS status were excluded from the analysis. Abbreviations: CI, confidence interval; ECOG PS, Eastern Cooperative Oncology Group performance status; EGFR, epidermal growth factor receptor; GERCOR, Groupe Coopérateur Multidisciplinaire en Oncologie; HR, hazard ratio; NLR, neutrophil-to-lymphocyte ratio; RMH, Royal Marsden Hospital; VEGF, vascular endothelial growth factor. Bold values indicate statistically significant findings; blank cells were excluded by backward LR.

## Data Availability

The data presented in this study are available on reasonable request from the corresponding author. The data is not publicly available due to ethical and privacy restrictions. The statistical code used for the C-index and bootstrap analyses in this study is available from the corresponding author upon reasonable request.

## Supplementary Materials

The following supporting information can be downloaded at: https://www.mdpi.com/article/10.3390/jcm15187149/s1. Table S1: Univariable and full clinical multivariable Cox regression analyses of factors associated with progression-free survival; Table S2: Univariable and full clinical multivariable Cox regression analyses of factors associated with overall survival; Table S3: Univariable Cox regression analysis of individual RMH score components for PFS and OS; Supplemental File: STROBE Statement—Checklist of Items to be Included in Reports of Cohort Studies [25].

## Abbreviations

CI	Confidence interval
CRC	Colorectal cancer
dMMR	Deficient mismatch repair
ECOG	Eastern Cooperative Oncology Group
EGFR	Epidermal growth factor receptor
GERCOR	Groupe Coopérateur Multidisciplinaire en Oncologie
HR	Hazard ratio
LDH	Lactate dehydrogenase
mCRC	Metastatic colorectal cancer
MMR	Mismatch repair
NLR	neutrophil-to-lymphocyte ratio
OS	Overall survival
PFS	Progression-free survival
RMH	Royal Marsden Hospital
VEGF	Vascular endothelial growth factor
