# Supplementary material for: Prognostic Value of the Royal Marsden Hospital Score in Patients with De Novo Metastatic Colorectal Cancer Receiving First-Line Systemic Therapy
Source: J Clin Med. 2026 Sep 15;15(18):7149. doi: 10.3390/jcm15187149 (PMC13607473; doi:10.3390/jcm15187149)
Supplement: Supplementary file 1 [file jcm-15-07149-s001.zip › STROBE_checklist.pdf]

STROBE Statement — Checklist of Items to be Included in Reports of Cohort Studies

Manuscript: Prognostic Value of the Royal Marsden Hospital Score in Patients with De Novo Metastatic Colorectal Cancer Receiving First-Line Systemic Therapy

Item	Section	STROBE Recommendation	Reported on / Location in Manuscript
**1a**	Title/Abstract	Indicate the study design with a commonly used term.	Abstract, Methods: "single-center retrospective cohort study" (also Methods 2.1)
**1b**	Title/Abstract	Provide an informative and balanced summary of what was done and what was found.	Abstract (structured: Background, Methods, Results, Conclusions)
**2**	Introduction – Background/rationale	Explain the scientific background and rationale for the investigation being reported.	Introduction, paragraphs 1–4
**3**	Introduction – Objectives	State specific objectives, including any prespecified hypotheses.	Introduction, final paragraph
**4**	Methods – Study design	Present key elements of study design early in the paper.	Methods 2.1, first sentence
**5**	Methods – Setting	Describe the setting, locations, and relevant dates, including periods of recruitment, exposure, follow-up, and data collection.	Methods 2.1 (Jan 2015–Jan 2026, single center); Methods 2.2 (data cutoff, June 11, 2026)
**6a**	Methods – Participants	Give the eligibility criteria and the sources and methods of selection of participants.	Methods 2.1 (inclusion/exclusion criteria); Figure 1 (flow diagram)
**6b**	Methods – Participants	For matched studies, give matching criteria and number of exposed/unexposed.	Not applicable (no matching was performed)
**7**	Methods – Variables	Clearly define all outcomes, exposures, predictors, potential confounders, and effect modifiers.	Methods 2.2 (variables collected; RMH score definition); Table 1
**8**	Methods – Data sources/measurement	For each variable of interest, give sources of data and details of methods of assessment; describe comparability of assessment methods if more than one group.	Methods 2.2 (retrospective extraction from archived files/electronic records; timing of laboratory measurements)
**9**	Methods – Bias	Describe any efforts to address potential sources of bias.	Methods 2.2 (exclusion of values obtained during active infection); Discussion, Limitations paragraph
**10**	Methods – Study size	Explain how the study size was arrived at.	Methods 2.1: all consecutive eligible patients treated during the study period were included; no separate sample-size calculation was performed, consistent with the retrospective design
**11**	Methods – Quantitative variables	Explain how quantitative variables were handled; describe grouping and why.	Methods 2.2–2.4 (RMH and GERCOR categorized per Table 1; NLR analyzed as a continuous variable)
**12a**	Methods – Statistical methods	Describe all statistical methods, including those used to control for confounding.	Methods 2.4 (multivariable Cox regression, backward likelihood ratio selection)
**12b**	Methods – Statistical methods	Describe any methods used to examine subgroups and interactions.	Methods 2.2/Results 3.2–3.3 (four-category RMH score analysis); Discussion, Limitations
**12c**	Methods – Statistical methods	Explain how missing data were addressed.	Methods 2.1 (patients with missing essential clinical or laboratory data were excluded); Results 3.1 (missing molecular data reported)

Item	Section	STROBE Recommendation	Reported on / Location in Manuscript
**12d**	Methods – Statistical methods (cohort)	If applicable, explain how loss to follow-up was addressed.	Methods 2.2 (patients without the event of interest were censored at the date of last follow-up)
**12e**	Methods – Statistical methods	Describe any sensitivity analyses.	Methods 2.4 / Results 3.2–3.3 (era-stratified sensitivity analysis: pre-2020 vs. 2020 onward)
**13a**	Results – Participants	Report the numbers of individuals at each stage of the study.	Figure 1 (flow diagram); Results 3.1 (n = 189 included)
**13b**	Results – Participants	Give reasons for non-participation at each stage.	Figure 1 (flow diagram); Methods 2.1 (exclusion criteria)
**13c**	Results – Participants	Consider use of a flow diagram.	Figure 1
**14a**	Results – Descriptive data	Give characteristics of study participants and information on exposures and potential confounders.	Results 3.1; Table 2
**14b**	Results – Descriptive data	Indicate the number of participants with missing data for each variable of interest.	Results 3.1 (BRAF, MMR, and HER2 status unavailable in a subset of patients)
**14c**	Results – Descriptive data (cohort)	Summarize follow-up time.	Results 3.2 (median follow-up 67.2 months, reverse Kaplan–Meier method)
**15**	Results – Outcome data	Report numbers of outcome events or summary measures over time.	Results 3.2 (166 progression events, 87.8%) and 3.3 (142 deaths, 75.1%)
**16a**	Results – Main results	Give unadjusted and, if applicable, confounder-adjusted estimates and their precision; clarify confounders adjusted for and why they were included.	Results 3.2–3.3; Tables 4 and 5 (univariable and multivariable HRs with 95% CI)
**16b**	Results – Main results	Report category boundaries when continuous variables were categorized.	Table 1 (RMH and GERCOR score definitions); Methods 2.2
**16c**	Results – Main results	If relevant, translate relative risk into absolute risk for a meaningful time period.	Median PFS/OS values by risk group are reported (Results 3.2–3.3) as an absolute-scale complement to hazard ratios
**17**	Results – Other analyses	Report other analyses done, e.g., subgroup analyses, interactions, and sensitivity analyses.	Results 3.2–3.3 (four-category RMH survival analysis; C-index comparisons among RMH, GERCOR, and NLR)
**18**	Discussion – Key results	Summarize key results with reference to study objectives.	Discussion, first paragraph
**19**	Discussion – Limitations	Discuss limitations of the study, taking into account sources of potential bias or imprecision; discuss both direction and magnitude of any potential bias.	Discussion, Limitations paragraph
**20**	Discussion – Interpretation	Give a cautious overall interpretation considering objectives, limitations, multiplicity of analyses, results from similar studies, and other relevant evidence.	Discussion, throughout (comparison with prior RMH, GERCOR, and NLR literature; clinical-perspective paragraph)
**21**	Discussion – Generalisability	Discuss the generalisability (external validity) of the study results.	Discussion, Limitations paragraph (single-center design; restriction to ECOG PS 0–1 and de novo disease)

Item	Section	STROBE Recommendation	Reported on / Location in Manuscript
**22**	Other information – Funding	Give the source of funding and the role of funders for the present study and, if applicable, for the original study on which the present article is based.	Funding statement (end of manuscript)

Note: This checklist is based on the STROBE Statement checklist for cohort studies (von Elm E, Altman DG, Egger M, et al. The Strengthening the Reporting of Observational Studies in Epidemiology (STROBE) statement: guidelines for reporting observational studies. Lancet. 2007;370(9596):1453-1457).
