# Supplementary material for: Prognostic Value of the Royal Marsden Hospital Score in Patients with De Novo Metastatic Colorectal Cancer Receiving First-Line Systemic Therapy
Source: J Clin Med. 2026 Sep 15;15(18):7149. doi: 10.3390/jcm15187149 (PMC13607473; doi:10.3390/jcm15187149)
Supplement: Supplementary file 1 [file jcm-15-07149-s001.zip › Tables S1-S3.pdf]

Supplementary Table S1.

Univariable and full clinical multivariable Cox regression analyses of factors associated with progression-free survival.

	Univariable analyses		Multivariable analyses	
**Variable**	**HR (95% CI)**	***p*-value**	**HR (95% CI)**	***p*-value**
Age (≥ 65 vs < 65 years)	1.31 (0.93–1.84)	0.117	1.21 (0.86–1.71)	0.277
ECOG PS (1 vs 0)	1.21 (0.89–1.65)	0.230	0.89 (0.64–1.25)	0.516
Tumor location		0.637		0.760
Right-sided colon	Reference		Reference	
Left-sided colon	0.83 (0.57–1.21)	0.343	0.86 (0.56–1.32)	0.485
Rectum	0.89 (0.59–1.34)	0.602	0.95 (0.60–1.51)	0.829
RAS mutation (mutant vs wild type)	1.22 (0.89–1.66)	0.208	1.26 (0.80–1.99)	0.315
Liver metastasis (yes vs no)	1.14 (0.80–1.62)	0.457	1.07 (0.71–1.60)	0.753
Peritoneal metastasis (yes vs no)	1.33 (0.92–1.93)	0.128	1.01 (0.67–1.53)	0.963
First-line chemotherapy (oxaliplatin vs irinotecan)	1.02 (0.71–1.45)	0.915	1.09 (0.75–1.58)	0.655
First-line targeted agent		**0.033**		**0.039**
No targeted therapy	Reference		Reference	
Anti-VEGF	0.53 (0.29–0.97)	**0.040**	0.43 (0.22–0.83)	**0.011**
Anti-EGFR	0.43 (0.22–0.81)	**0.010**	0.49 (0.25–0.98)	**0.045**
RMH risk group (high vs low)	2.13 (1.50–3.02)	**< 0.001**	2.24 (1.53–3.26)	**< 0.001**

The full multivariable Cox regression model included 180 patients and 161 events; 9 patients with unknown RAS status were excluded from the analysis. **Abbreviations:** CI, confidence interval; EGFR, epidermal growth factor receptor; HR, hazard ratio; RMH, Royal Marsden Hospital; VEGF, vascular endothelial growth factor. Bold values indicate statistically significant findings.

Supplementary Table S2.

Univariable and full clinical multivariable Cox regression analyses of factors associated with overall survival.

	**Univariable analyses**		**Multivariable analyses**	
**Variable**	**HR (95% CI)**	***p*-value**	**HR (95% CI)**	***p*-value**
Age (≥ 65 vs < 65 years)	1.62 (1.16-2.28)	**0.005**	1.64 (1.12–2.40)	**0.011**
ECOG PS (1 vs 0)	1.45 (1.03–2.03)	**0.032**	1.06 (0.73–1.53)	0.743
Tumor location		0.518		0.138
Right-sided colon	Reference		Reference	
Left-sided colon	0.85 (0.57–1.27)	0.447	1.04 (0.63–1.70)	0.867
Rectum	0.77 (0.49–1.20)	0.256	0.67 (0.38–1.16)	0.159
RAS mutation (mutant vs wild)	1.48 (1.05–2.07)	**0.023**	2.08 (1.21–3.57)	**0.008**
Liver metastasis (yes vs no)	0.88 (0.62–1.27)	0.515	0.69 (0.44–1.06)	0.096
Peritoneal metastasis (yes vs no)	1.49 (1.01–2.19)	**0.044**	0.97 (0.61–1.53)	0.902
First-line chemotherapy (oxaliplatin vs irinotecan)	0.87 (0.59–1.28)	0.497	0.72 (0.47–1.10)	0.132
First-line targeted agent		0.074		**0.011**
No targeted therapy	Reference		Reference	
Anti-VEGF	0.54 (0,29–1,00)	0.051	0.39 (0.19–0.79)	**0.009**
Anti-EGFR	0.46 (0,24–0,89)	**0.023**	0.78 (0.38–1.60)	0.515
RMH risk group (high vs low)	3.01 (2.08–4.36)	**< 0.001**	3.40 (2.23–5.18)	**< 0.001**

The full multivariable Cox regression model included 180 patients and 136 deaths; 9 patients with unknown RAS status were excluded from the analysis. The difference in the statistical significance of RAS between the reduced and full OS models likely reflects the different covariate sets included in the two models. **Abbreviations:** CI, confidence interval; ECOG PS, Eastern Cooperative Oncology Group performance status; EGFR, epidermal growth factor receptor; HR, hazard ratio; RMH, Royal Marsden Hospital; VEGF, vascular endothelial growth factor. Bold values indicate statistically significant findings.

Supplementary Table S3.
Univariable Cox regression analysis of individual RMH score components for PFS and OS

	PFS		OS	
**RMH component**	**HR (95% CI)**	***p*-value**	**HR (95% CI)**	***p*-value**
Albumin <3.5 g/dL	1.64 (1.17–2.31)	**0.005**	2.46 (1.72–3.53)	**<0.001**
LDH >250 U/L	1.36 (1.00–1.86)	0.050	1.39 (0.99–1.93)	0.055
≥3 metastatic organs	2.03 (1.40–2.96)	**<0.001**	2.42 (1.60–3.65)	**<0.001**

Abbreviations: CI, confidence interval; HR, hazard ratio; LDH, lactate dehydrogenase; OS, overall survival; PFS, progression-free survival; RMH, Royal Marsden Hospital. Bold values indicate statistically significant results ($p < 0.05$).
